# Hypoxia and Integrin-Mediated Epithelial Restitution during Mucosal Inflammation

**DOI:** 10.3389/fimmu.2013.00272

**Published:** 2013-09-11

**Authors:** Bridie J. Goggins, Ciaran Chaney, Graham L. Radford-Smith, Jay C. Horvat, Simon Keely

**Affiliations:** ^1^School of Biomedical Sciences and Pharmacy, University of Newcastle, Newcastle, NSW, Australia; ^2^Hunter Medical Research Institute, New Lambton, NSW, Australia; ^3^Royal Brisbane and Women’s Hospital, Brisbane, QLD, Australia; ^4^Queensland Institute for Medical Research, Brisbane, QLD, Australia

**Keywords:** hypoxia, integrins, epithelial cells, wound healing, mucosal immunity, HIF-1a

## Abstract

Epithelial damage and loss of intestinal barrier function are hallmark pathologies of the mucosal inflammation associated with conditions such as inflammatory bowel disease. In order to resolve inflammation and restore intestinal integrity the mucosa must rapidly and effectively repair the epithelial barrier. Epithelial wound healing is a highly complex and co-ordinated process and the factors involved in initiating intestinal epithelial healing are poorly defined. In order for restitution to be successful there must be a balance between epithelial cell migration, proliferation, and differentiation within and adjacent to the inflamed area. Endogenous, compensatory epithelial signaling pathways are activated by the changes in oxygen tensions that accompany inflammation. These signaling pathways induce the activation of key transcription factors, governing anti-apoptotic, and proliferative processes resulting in epithelial cell survival, proliferation, and differentiation at the site of mucosal inflammation. In this review, we will discuss the primary processes involved in epithelial restitution with a focus on the role of hypoxia-inducible factor and epithelial integrins as mediators of epithelial repair following inflammatory injury at the mucosal surface.

## Introduction

The successful healing of a mucosal wound requires the inter-related processes of inflammation, proliferation, granulation tissue formation, and tissue remodeling (1). These are highly regulated and over-lapping events with environmental stimulus from one event, dictating progression to the next (2). One important aspect of mucosal wound healing is the role of oxygen sensing in the tissue repair process, given the dramatic changes in tissue oxygen tension during inflammation and wounding, where oxygen tensions may decrease 10-fold at a wound site (3). Despite these changes most inflammatory wounds resolve and tissue homeostasis is restored. Thus, wounded and inflamed tissues may adapt to reduced oxygen availability at an inflammatory wound and retain the ability to repair, despite tissue hypoxia. Much of what we know about wound healing processes is derived from studies in dermal healing and there is relatively little known about mechanisms of mucosal wound healing, particularly at the intestinal mucosa, where normal oxygen tensions are low (4).

## Mucosal Inflammation and Hypoxia

In the early stages of the initial mucosal insult, intestinal wounds are almost devoid of oxygen (3). This is a result of both the vascular damage occurring with injury and increased cellular oxygen demand at the wound. Infiltrating immune cells generate superoxide, combating infection, but greatly increasing oxygen demand (3, 5). In addition reparative processes such as cell proliferation and collagen production increase oxygen demand in the mucosal environment (6). Thus, the initial inflammatory response to mucosal damage promotes a state of chronic hypoxia within the microenvironment of the wound. This “inflammatory hypoxia” has been elegantly demonstrated in murine models of colitis. For instance, the 2,4,6-trinitrobenzenesulfonic acid (TNBS) model of murine colitis has been widely utilized to model inflammatory bowel diseases (IBDs) (7). Histologically these animals demonstrate profound vasculitis of the small submucosal vessels associated with mucosal inflammation, similar to observations in human tissues (8, 9). The chronic hypoxia and inflammation within the intestinal mucosa is associated with angiogenesis, further enhancing influx of inflammatory cells and endothelial dysfunction (10). Associated upregulation of collagen synthesis increases the risk of fibrosis, a key feature of chronic inflammatory disease potentially driven by chronic tissue hypoxia (11). Tissue hypoxia associated with inflammation has been demonstrated in animal models, through utilization of the characteristic reduction and binding of 2-nitroimidazole compounds, such as pimonidazole and EF5, to cellular thiol-containing proteins oxygen levels below 10 mmHg (12).

Animals with TNBS-induced colitis demonstrated dramatic levels of nitroimidazole retention associated with colitic lesions, both in superficial and in deeper submucosal regions of the mucosa (13, 14). This is in marked contrast to the superficial retention of nitroimidazoles observed in healthy animals. These findings, demonstrated in several other animal models, indicate that mucosal inflammation, such as that associated with models of mucosal inflammation likely result in significant tissue hypoxia, predominantly within the epithelium.

## Molecular Signaling by Hypoxia

Oxygen is a key component in the generation of metabolic energy for all eukaryotic cells (15). Fluctuations in tissue oxygen supply (hypoxia) are common physiologic and pathophysiologic occurrences. These include frank vascular occlusion such as those occurring with stroke, tissue fibrosis, and the microvascular breakdown associated with chronic inflammation which also results in localized tissue hypoxia/ischemia. Alternatively, diminished oxygen delivery to tissues may occur in shock, hypotension, or in cases where the oxygen carrying capacity of blood is compromised [e.g., chronic obstructive pulmonary disease (COPD), carbon monoxide poisoning] (16, 17). Thus, mammalian cells have evolved compensatory mechanisms to adapt to tissue hypoxia (18). One such mechanism is the oxygen-sensing molecule; hypoxia-inducible factor (HIF), a transcription factor which functions as a global mechanism for adaptation to hypoxia (19).

## Hypoxia-Inducible Factor Regulation and Cellular Oxygen Sensing

**Figure 1:**
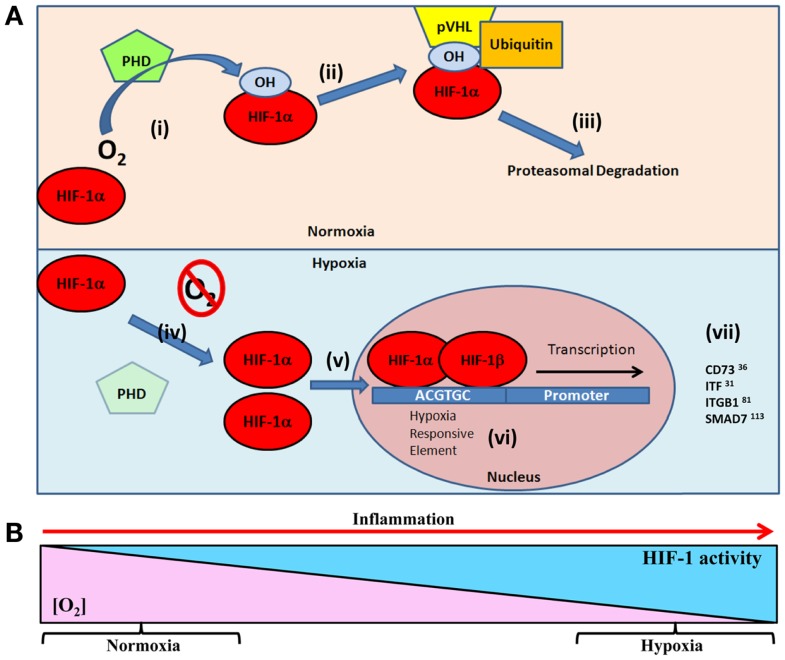
**(A)** Oxygen-dependent regulation of HIF-1α targets in epithelial restitution. Under normal oxygen tensions (normoxia) (i) prolyl hydroxylase (PHD) enzymes hydroxylate the HIF-1α subunit resident in the cellular cytoplasm (26). Hydroxylated HIF-1α facilitates, (ii) the binding of von Hippel–Lindau protein (pVHL) and subsequent recruitment of the ubiquitin ligase complex, (iii) targeting HIF-1α for 26S proteasomal degradation (28). Under conditions of reduced oxygen (hypoxia) (iv) the lack of oxygen substrate for PHD prevents hydroxylation of HIF-1α leading to (v) cytoplasmic accumulation and translocation to the cell nucleus (29). (vi) HIF-1α dimerizes with HIF-1β and binds to hypoxia responsive elements (5′-ACGTGC-3′) in the promoter of target genes (21, 30). (vii) This leads to transcription of HIF target genes involved in epithelial restitution. **(B)** As tissue oxygen levels gradually decline, HIF stabilization increases. This results in graded HIF stabilization during progressive hypoxia (31), such as the progression of inflammation.

Hypoxia-inducible factor is a central regulatory transcription factor for hypoxia-induced gene expression, and serves as a sensitive and selective indicator of hypoxia (20–22). HIF is a heterodimeric nuclear protein made up of an α oxygen regulated and constitutively expressed β subunit (23, 24). Under conditions of normal tissue oxygen tensions (normoxia), the α subunit is continuously synthesized, and degraded through a cascade of events. The prolyl residues (402 and/or 564) on the α subunit undergo oxygen-dependent hydroxylation by the prolyl-4-hydroxylase (PHD) enzymes. PHDs, principally prolyl hydroxylase-2 (PHD2), target oxygen, and α-ketoglutarate as substrates to catalyze a dioxygenase reaction (22, 25), which facilitates hydroxylation of the hypoxia-inducible factor-1α (HIF-1α) subunit (26). This leads to binding of the von Hippel–Lindau (VHL) protein, which allows the recruitment of the ubiquitin ligase complex (22, 27) and targets HIF-1α for proteasomal degradation by the 26*S* proteasome (28). However, during periods of reduced oxygen availability (hypoxia), PHD2 activity is reduced due to substrate (oxygen) limitations. This allows stabilization of HIF-1α within the cytoplasm of the cell and translocation to the nucleus for dimerization with the HIF-1β subunit (29). Dimerization forms a transcriptionally functional HIF-αβ dimer, which then binds to cis-acting hypoxia response elements (HREs) in the promoter of target genes and recruits co-activator proteins (Figure 1A). As a result of this cascade, transcription of HIF target gene sequences to mRNA is increased (21, 30). However, this is not an all or nothing response, and HIF-1α stabilization is gradual and graded over the progression from mild to chronic hypoxia (31) (Figure 1B).

Until very recently, most work with HIF focused on understanding the basic mechanisms by which HIF acts as a key mediator of the cellular hypoxic response, particularly in the context of carcinogenesis (17, 32). Solid tumors have been demonstrated to form hypoxic cores and adapt to this oxygen deficiency in order to maintain a proliferative state. However, recent studies reveal a potentially central role for HIF in endogenous protective and restorative pathways within a variety of inflammatory diseases, including respiratory distress syndrome, retinitis, diabetes, and arthritis (17).

## HIF and Adaption to Mucosal Inflammation

Activation of HIF-1α due to the unavailability of oxygen has been widely shown to promote adaption to inflammation, primarily through an increase in mucosal barrier protection (4, 33, 34). Key to this protective response is the induction of genes involved in non-classical epithelial barrier function. These include genes that regulate the integrity of the mucous-gel layer; Mucin 1 and 3 (MUC1 and MUC3) (34, 35) and intestinal trefoil factor (ITF) (33), the epithelial xenobiotic drug efflux pump; (multi drug resistance protein 1, MDR1; P-glycoprotein) (36), leukocyte trafficking and clearance; CD55 (decay accelerating factor) (37), and cellular energy metabolism; CD73 (ecto-5′-nucleotidase) (38), and the adenosine A2B receptor (39). Thus, genes induced by HIF-1α support overall tissue integrity and include target proteins necessary for cellular, whole tissue, and whole animal adaptive responses to hypoxia (40, 41).

## HIF and Mucosal Healing

Hypoxia-inducible factor regulates a diverse number of genes, many of which feeding back into processes critical for wound healing (31). While HIF signaling allows the tissue to adapt to, and protect against, inflammatory hypoxia, HIF also regulates the expression of genes that drive angiogenesis. As inflammatory damage to the tissue is a key driving factor in tissue hypoxia, it is unsurprising that HIF adaptive responses include restoration of the vascular oxygen supply. In particular, HIF regulates the expression of vascular endothelial growth factor A (VEGFA) and angiopoietins, which drive angiogenesis through endothelial mitosis and migration (42–45). As evidence of the role of HIF in wound healing, over-expression of HIF-1α improves wound healing in mouse models of diabetes (29, 46), a condition where impaired healing may lead to complications such as diabetic foot (47). Conversely suppression of HIF-1α expression results in dysfunctional wound healing and defective vascularization (48).

Hypoxia-inducible factor also regulates the induction of VEGF receptor Flt-1 (49, 50) and a range of vasomotor peptides, such as adrenomedullin (51) and endothelin-1 (52) which act to fine tune the angiogenic response, underpinning the importance of HIF signaling in the regulation of angiogenesis. The potential risks associated with angiogenesis in chronic inflammatory disease states such as CD include formation of a dysfunctional new vessel architecture and further recruitment of inflammatory cells. In the absence of fine tuning of the angiogenic response that remains functional in acute intestinal inflammation such as infectious colitis, the responses seen in CD lead to fibrosis, and the need for bowel resection (53).

In contrast to angiogenesis, little is known about how hypoxia and HIF signaling directly influences mucosal, epithelial wound healing at the molecular level. Moreover, while there is evidence of the importance of HIF in regulating keratinocyte re-epithelialization (54), there is far less understanding of how transcriptional amplification by hypoxia might be important in initiating mucosal wound healing responses.

## Epithelial Wound Healing

Early in the healing process, epithelial cells adjacent to the mucosal wound lose polarity and convert into a migratory phenotype (55). The depolarized epithelial cells rapidly migrate into the denuded area and line the underlying matrix in order to re-establish a protective barrier (56). Once the barrier has been restored, epithelial cell proliferation begins and enterocyte numbers increase to resurface the wounded area (57). Proliferation occurs hours to days after injury, usually in the crypts near the damaged mucosal area. Finally, proliferation progenitor epithelial cells must differentiate into a specific lineage subset. Functionally, these encompass absorptive enterocytes or one of three secretory lineages cells (goblet, enteroendocrine, and paneth cells) (58). Once differentiated, IECs can restore the functional activities of the epithelial layer and intestinal homeostasis resumes (55).

Damage to the intestinal epithelial layer is a common pathology of the inflammation associated with diseases such as IBD (56). In order to suppress inflammation and restore normal intestinal homeostasis the mucosa must activate rapid repair mechanisms and restore epithelial defenses (59, 60). Even the most superficial injuries to the epithelium result in epithelial destruction and require healing (61). Successful epithelial repair requires a balance between epithelial restitution, proliferation, and differentiation within and adjacent to areas of mucosal damage (55, 62). Thus, for the resolution of inflammation to occur, the tissue must first halt the influx of luminal antigens through the damaged epithelium. Accordingly, the first phase of the wound healing process is restitution of the epithelial barrier.

## Epithelial Restitution

Restitution is the rapid migration of epithelial cells adjacent to the wound/injury in order to reseal the damaged area. Migrating cells achieve restitution through a sequence of transient adherence to the extra-cellular matrix. This adherence is achieved via a collection of specialized basal structures that evolve from focal complexes followed by focal adhesions to fibrillar adhesions (63–67). Rapid migration of the epithelial cells adjacent to the wound, at the wound edge, marks the initiation of restitution, and this begins within minutes to hours of the injury occurring (56, 68). Epithelial cells surrounding the wound have the ability to rapidly migrate due to the loss of columnar polarity. These cells undergo extensive reorganization of their actin cytoskeleton (55, 69), losing their microvilli, and apical/basolateral orientation to adopt a flat morphology. The cells then re-polarize to induce migration, with polarization now defined from leading to trailing edge (70–72). Migration is dependant on F-actin-rich protrusions called lamellipodia at the leading edge, which enable transient adherence to the underlying matrix at focal adhesion complex sites (73, 74). This change in polarization and shape allows the cells to migrate rapidly to the injury site and attach via focal contacts (75), restoring barrier integrity (56, 68, 76). Restitution is a very rapid process and enables reconstitution of epithelial continuity much faster than could be achieved through proliferation of cells alone. However, proliferation is still required to ultimately restore the mucosal surface. Restitution is the central component to epithelial healing regardless of the cause or extent of the injury, as restitution ensures epithelial continuity is re-established (77).

## HIF-Mediated Epithelial Restitution

**Figure 2:**
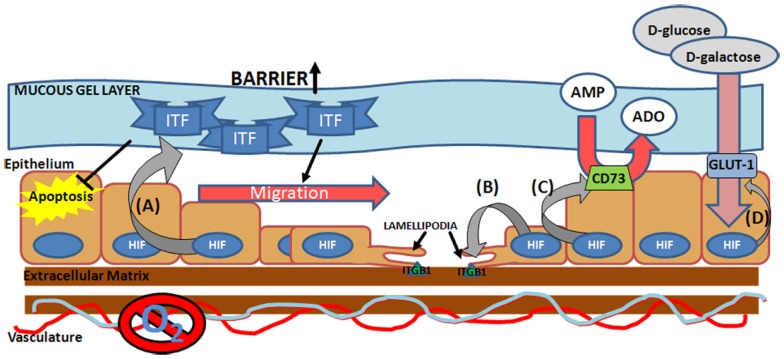
**Hypoxia-inducible factor-mediated pro-restitution pathways**. Hypoxia-inducible factor (HIF) activation at the site of the mucosal wound leads to induction of **(A)** epithelial intestinal trefoil factor (ITF) (33), which acts to increase mucosal barrier function, suppress epithelial apoptosis (127), and drive epithelial migration (80), **(B)** integrin β_1_ (ITGB1) (84), a lamellipodia protein critical for epithelial cell migration across the extra-cellular matrix, **(C)** ecto-5′-nucleotidase (CD73) (38), which facilitates conversion of AMP into adenosine (ADO), and **(D)** glucose transporter 1 (GLUT-1) (7, 8), which facilitates the transport of D-glucose and D-galactose across the plasma membrane.

In order to facilitate repair of the epithelial barrier, HIF directly targets a number of critical components for the epithelial wound healing process including energy metabolism and cell migration, both important processes in restitution (Figure 2). Induction of CD73 and glucose transporter 1 (GLUT-1), allow the cells to maintain energy metabolism in the hypoxic microenvironment of the mucosal wound (38, 78). Pre-epithelial barriers are augmented through the induction of mucins and ITF (33). ITF is a particularly noteworthy factor in mucosal wound healing, as it not only augments the barrier, through increased mucosal integrity via interactions with mucin glycoproteins (79), but also facilitates epithelial restitution. Both apically secreted and exogenous ITF accelerate epithelial cell migration into the wound area through pathways independent of transforming growth factor-β (TGF-β) signaling at the basolateral interface (68, 80). In addition ITF can prolong epithelial cell life at the site of a wound, through inhibition of apoptosis (81). Thus HIF-mediated induction of ITF, not only acts to protect a wounded mucosal surface, but also modulates epithelial restitution. However, a double-blind, randomized, placebo-controlled study, to examine the efficacy of supplementing conventional treatments with recombinant ITF for the treatment of mild-to-moderate ulcerative colitis, did not reveal any additional benefit above that of conventional therapies alone (82). This could be due to the enema method of ITF delivery, which may not facilitate sufficient ITF-epithelial interactions to mediate restitution. The study did not measure mucosal responses to local delivery of ITF to determine or confirm whether it was biologically active in the disease state in human subjects. Further studies will need to address this.

Hypoxia-driven motility is associated with increased expression of lamellipodia proteins, increased expression of collagenase and decreased expression of laminin-5, the locomotion brake for keratinocytes (83). Our own studies have identified HIF-1α stabilization as a key promoter of integrin β_1_ (ITGB1), a critical mediator of lamellipodia binding, during wound healing in IBD (84). Here we demonstrated direct binding of HIF to the ITGB1 promoter, leading to increased expression of functional β_1_ integrin. In TNBS models of colitis, mucosal ITGB1 expression correlated directly with disease severity, revealing a correlation between mucosal hypoxia and ITGB1 expression *in vivo*. Interestingly, site-directed mutagenesis of the hypoxia responsive element (HRE) on the ITGB1 promoter did not completely abolish the HIF response, suggesting that secondary mechanisms may also be involved, for instance inhibitor of DNA binding-1 (Id-1) has been shown to induce ITGB1 in a HIF-dependent manner and Qiu et al. have speculated on whether Id-1 may regulate the ITGB1 promoter directly (85). Nevertheless, in subsequent studies examining the role of HIF-1α stabilization in mucosal response to colitis in murine models, we have demonstrated that stabilization of HIF-1α through prolyl hydroxylase inhibition (PHDi) results in earlier and increased epithelial ITGB1 expression, concurrent with accelerated mucosal healing and restitution of epithelial barrier function (86). Furthermore, the protective effect of the pharmacological HIF stabilization by PHDi is lost in conditional epithelial HIF deficient animals. Further evidence for the role of HIF in integrin-mediated wound healing has been shown in dermal healing models. HIF-1α silencing, led to decreased expression of Lamanin-322, α6 integrin and β_1_ integrin, and this was associated with impaired epidermal healing and dermis formation in both murine models and human keratinocyte cultures (87). This implicates an importance for HIF in aging and wound healing processes. Overall, these data suggest direct roles for both HIF-1α and ITGB1 in mediated mucosal wound healing.

## Integrins as Mediators of Epithelial Wound Healing

A key factor in the co-ordination of restitution is the ability of cells to adhere to, and interact with the extra-cellular matrix. Integrins are critical mediators of these interactions and facilitate epithelial migration into the denuded mucosal wound. Integrins are a family of cell adhesion receptors responsible for mediating both cell-substratum and cell–cell adhesion (88). They exist as heterodimeric glycoproteins consisting of non-covalently bonded α and β subunits (89–91). Integrins provide essential links between the extra-cellular environment and intracellular signaling pathways. This makes them key regulators of cell behaviors such as cell survival, apoptosis, differentiation, migration, and transcriptional regulation (92), thus integrins are critical for processes in development, immune function, and wound healing.

In the context of wound healing and epithelial restitution Lotz et al. showed the functional importance of integrin heterodimers containing a β_1_-subunit, in particular α_6_β_1_ and α_3_β_1_ integrins in epithelial wound restitution (93). In T84 epithelial wounding models, cell migration was shown to be integrin-dependent, regulated by the expression of localized, specific integrins, and their cell matrix protein ligands. Monoclonal antibodies directed against functional epitopes on α_3_ and β_1_ integrins were found to inhibit wound closure completely, while functional blockade of α_6_ integrin also significantly inhibited wound closure. These heterodimers are differentially expressed within the process of restitution, with α_6_β_1_ integrin increased along the lateral surfaces of migrating cells, while α_3_β_1_ integrin expression localized to flattened cell surfaces and along wound and colony perimeters (93). These studies suggest a fundamental role of integrins, particularly α_3_β_1_ and α_6_β_1_, in epithelial restitution.

## Inflammatory Signaling and Integrin-Mediated Restitution

Epithelial injury observed in patients with IBD is associated with the infiltration of inflammatory cells to the mucosa, which triggers an inflammatory cascade in the tissue causing the release of proinflammatory cytokines and, often, further tissue injury (94, 95). Secreted inflammatory cytokines can also directly influence the progression of epithelial restitution (96). For instance, interferon-γ (IFN-γ) has been identified as a key proinflammatory cytokine in IBD, with elevated levels observed in the mucosa of IBD patients (97). While extensive investigations have been conducted on the effects of IFN-γ on epithelial intercellular junctions and barrier properties (94, 98–100), there is a paucity of studies characterizing the effect of IFN-γ on the wound healing process. To address this, Tong et al. investigated the influence of IFN-γ on intestinal epithelial wound closure (96), examining epithelial cell migration *in vitro*. IFN-γ demonstrated clear inhibitory effects on epithelial migration, causing dysfunction of the F-actin-rich lamellipodia protrusions at the leading edge of the migrating cell. No difference in the average number of lamellipodia at the leading edge of cells was found between control and IFN-γ-treated monolayers (96). As IFN-γ is known to drive pathology in a number of mucosal inflammatory diseases, these findings may explain the impaired wound healing observed in mucosal disease, where IFN-γ alters lamellipodia formation and subsequently impairs cell migration.

Attachment of lamellipodia occurs at focal adhesion complex sites, and key components of these focal adhesion sites are integrin heterodimers (55). As migrating cells move, continuous attachments are formed to the extra-cellular matrix at the leading edge of the cell, in synchrony with rear edge detachments until the wound is resealed by intercellular focal contacts (73). Integrins contribute to this process through cycles of exocytosis and endocytosis of surface bound integrins. Integrin heterodimers are transported via endocytic vesicles to the cell surface at the leading edge where they can form new focal complexes (101, 102). The mechanism of IFN-γ mediated lamellipodia dysfunction appears to involve several key focal adhesion proteins. For instance, IFN-γ suppresses expression of vinculin, focal adhesion kinase, and paxillin (96). IFN-γ also reduces the deposits of intracellular β_1_ integrin in focal adhesions at the leading edge of migrating epithelial cells, while reducing the number of β_1_ integrin containing cellular vesicles overall. This change in integrin distribution is not a result of accelerated degradation or loss of integrin protein, but rather accelerated endocytosis of membrane integrins. Migrating cells treated with IFN-γ-shown broadly distributed clusters of integrins throughout the cell, rather than the accumulation of β_1_ integrin observed at the leading edge of the migrating control cells (96). These studies further highlight the fundamental role of integrin β_1_ in the wound healing process and the importance of integrin β_1_ localization at leading edge focal complexes and vesicular transport of β_1_ for cell migration and movement.

Studies by Glover et al. (103) have further characterized the pathogenic contribution of IFN-γ to inflammatory diseases such as IBD. Inflammation is associated with dramatic shifts in tissue metabolism due to immune cell recruitment to inflammatory wounds or lesions (104). Hypothesizing that inflammatory cytokine signaling may input into the hypoxic response to mucosal inflammation, Glover et al. investigated the effect of inflammatory mediators on HIF regulation in intestinal epithelial monolayers. Investigating a host of common inflammatory cytokines, including TNF-α, IL-4, PGE_2_, and IFN-γ in both normoxic and hypoxic cultures, IFN-γ demonstrated the ability to significantly repress HIF-1 transcriptional targets in both normoxic and hypoxic conditions. In contrast, HIF-1α mRNA expression showed moderate increases in expression in response to IFN-γ and further investigation demonstrated that attenuation of HIF activity is the result of selective repression of HIF-1β.

While HIF responses drive expression of both protective and reparative pathways, the expression of HIF-1α is concurrent with chronic mucosal inflammation, suggesting that in chronic inflammatory diseases such as IBD, the HIF response is not always sufficient to promote restitution. This may in part, be due to increased levels of mucosal IFN-γ associated with chronic inflammation (103). *In vitro*, IFN-γ was shown to repress the expression of the HIF-1β and dextran sodium sulfate (DSS) murine models of IBD showed an inverse correlation between IFN-γ and HIF-1β expression. This result is surprising, in that HIF-α subunits are generally considered to be the regulated components of HIF signaling, while HIF-β subunits are regarded as constitutively expressed (23). As many studies only examine the expression of HIF-α isoforms, the study by Glover et al. may offer a critically important explanation as to why chronic inflammation progresses despite the stabilization of HIF-1α, given that IFN-γ is involved in the pathogenesis of many mucosal diseases (105–107). In particular, inhibition of HIF signaling by IFN-γ could, hypothetically, significantly impair mucosal healing, through reduced expression of a number of cellular proteins, such as β_1_ integrin, critical for epithelial restitution, and wound healing.

## Integrins and TGF-β-Mediated Pathways

Transforming growth factor-β is a pleiotropic cytokine and is critical to the regulation of cellular events involved in wound healing, including cell differentiation, proliferation, epithelial-mesenchymal transition, and cell migration. There is a strong degree of cross-talk between hypoxia and TGF-β (108), particularly the TGF-β_1_ isoform. Hypoxia has been shown to increase the transcription of TGF-β_1_ in dermal fibroblasts (109), while TGF-β_1_ may stabilize HIF-1α through selective inhibition of PHD2 (110). This inhibition is achieved through the downregulation of PHD2 gene expression via SMAD dependent pathways (111). SMAD proteins are intracellular TGF-β signal transducers that mediate the interaction between TGF-β receptor ligands and downstream nuclear responses (112). For instance, at the site of a wound, TGF-β_1_ mediated activation of SMAD2/3 complexes and subsequent interaction with SMAD4 leads to the formation of a SMAD transcription factor which drives cellular responses toward re-epithelialization (113–115).

**Figure 3:**
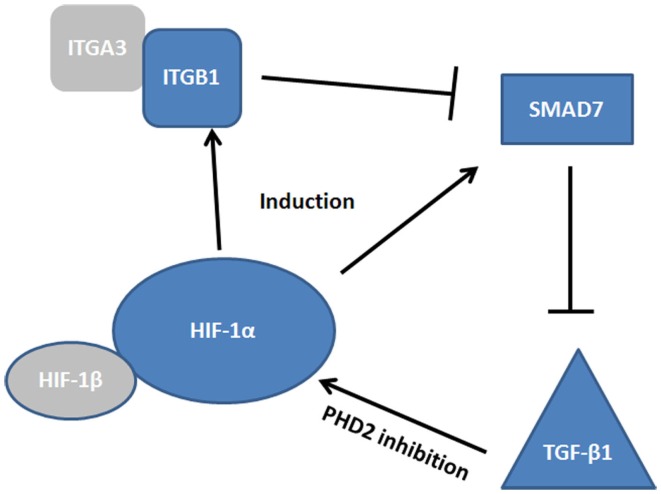
**Convergence of HIF, TGF-β, and SMAD pathways**. The complexity of the signaling cascade by which HIF regulates integrin β_1_ (ITGB1) and SMAD7 induction. At the site of the wound α3β_1_ integrin inhibits SMAD7, promoting TGF-β_1_ induction, which promotes restitution. How these factors interplay may be critical to our understanding of epithelial wound healing.

While few studies have been conducted in the context of mucosal inflammation and healing, there is evidence to suggest convergence of HIF, TGF-β, and SMAD pathways in the co-ordinated regulation of epithelial restitution (Figure 3). For instance, studies in the hypoxic microenvironment of solid tumors have identified SMAD7 as a HIF-1α responsive gene (116). SMAD7 has been shown to be a potent inhibitor of TGF-β_1_ (116, 117) and thus may prevent TGF-mediated cell proliferation and anti-inflammatory signaling. However, SMAD7, is itself inhibited by integrin signaling, specifically by epithelial integrin heterodimers containing an integrin β_1_ (ITGB1) subunit (118). Reynolds et al. demonstrated that α3β_1_ integrin heterodimers inhibited SMAD7 and enhanced cutaneous re-epithelialization in murine models of wound healing (118). As both ITGB1 and SMAD7 are HIF responsive, it is feasible that they represent co-dependent modulators of TGF-β_1_ mediated wound healing. α3β_1_ integrins are expressed in depolarized intestinal epithelial cells, particularly around the wound edge (93), thus induction of these integrins may act to inhibit SMAD7, promote TGF-β_1_ signaling, and initiate intestinal epithelial wound closure. Interestingly, SMAD7 is overexpressed in the inflamed mucosa of IBD patients (117) and targeting of SMAD7 has shown efficacy in mouse models of colitis (119), while ITGB1 single nucleotide polymorphisms have been identified as a risk factor in IBD (120). Thus, dysfunction of the pathway by which HIF-induced ITGB1 inhibits SMAD7, and the subsequent elevation of TGF-β_1_, may lead to the progression of chronic inflammation instead of mucosal wound healing. Importantly, TGF-β_1_ plays a role in the pathogenesis of intestinal fibrosis in Crohn’s patients (95) and TGF-β_1_ codon 25 variants are associated with structuring (121). Whether this polymorphism represents a dysfunction ins SMAD7/ITGB1/TGF-β_1_ signaling is unknown, but as HIF-mediated ITGB1 drives fibroblast-collagen contraction in vitro, any dysfunction in TGF-β_1_ signaling is likely to interplay in this pathway.

We may also consider that TGF-β_1_ acts to “fine tune” the HIF-1α response, as exogenous TGF-β_1_ enhances HIF-1α expression in hypoxic cells, while also increasing HIF-1α stabilization in normoxic conditions. As TGF-β_1_ does not affect transcription of HIF-1α itself, nor decrease degradation, it appears that this interplay occurs at the level of HIF-1α translation. This may occur independently of hypoxia, thus it is possible that TGF-β_1_ may act to “prime” the HIF response (122) in a manner similar to HIF-priming vasopeptides such as adrenomedullin (51).

## Summary

While a role for oxygen in mediating wound healing has been recognized for decades (6, 123, 124), the importance of cellular oxygen sensing in cellular adaptive and reparative pathways is a relatively new area (3, 16, 125). Given the rapidly changing oxygen tensions in the mucosal wound, the role for hypoxia responsive pathways in processes such as epithelial restitution is unsurprising. Hypoxia seems to independently regulate several critical drivers of epithelial restitution that subsequently exhibit a high degree of interplay. The interactions between HIF, β_1_ integrin heterodimers, SMAD7, and TGF-β are complex and have not been fully elucidated. Crucially, much of our knowledge of these pathways come from models of dermal wound healing, where basal oxygen levels are markedly higher than that of intestinal mucosal tissues (126).

Therapeutically, wound healing pathways are an attractive target for mucosal disease. For instance, despite the successes of immunomodulators in the maintenance of IBD, up to 70% of IBD patients still require surgery to remove tissue damaged by repeated cycles of inflammatory damage and improper healing. Therapies aimed at modulating the healing process may reduce the need for these surgeries. Further elucidation of the pathways driving mucosal wound healing are therefore critically important, and may open the door for improved therapeutic strategies for the management of mucosal inflammatory disease.

## Conflict of Interest Statement

The authors declare that the research was conducted in the absence of any commercial or financial relationships that could be construed as a potential conflict of interest.
